# Effect of 32-Weeks High-Intensity Interval Training and Resistance Training on Delaying Sarcopenia: Focus on Endogenous Apoptosis

**DOI:** 10.3389/fphys.2022.811369

**Published:** 2022-04-28

**Authors:** Hao Su, Tianhao Wen, Dongsen Liu, Jia Shao, Lei Zhao, Qi Gao

**Affiliations:** ^1^ Department of Exercise Biochemistry, Beijing Sport University, Beijing, China; ^2^ Military Common Subject Teaching and Research Section, PLA Rocket Force University of Engineering, Xi’an, China; ^3^ School of Sport Science, Beijing Sport University, Beijing, China; ^4^ Sport physical therapy and therapeutic exercise, sports health, Beijing Sport University, Beijing, China; ^5^ China Institute of Sport and Health Science, Beijing Sport University, Beijing, China; ^6^ Department of Exercise Physiology, Beijing Sport University, Beijing, China

**Keywords:** aging, sarcopenia, HIIT, resistance exercise, endogenous apoptosis

## Abstract

Sarcopenia caused by aging is an important factor leading to a decline in the quality of life of older people. Apoptosis in muscle atrophy accelerates the process of muscle loss in older populations. The present study aimed to investigate the effects of 32 weeks of high-intensity interval training (HIIT) and resistance training (RT) on the skeletal muscle-related indices and provide a theoretical basis for regulating the mitochondrial-mediated pathway to delay sarcopenia. We randomly selected 10 from eight-month-old male SD rats (*N* = 130) as the baseline group; after 1 week of adaptive feeding, the rats were sacrificed. The remaining rats were randomly assigned to one of three groups: control group (C, *N* = 40, natural aging for 32 weeks), HIIT group (H, *N* = 40, performed six loops of 3 min at 90% and 3 min at 50% VO2 max speed treadmill running, with 5 min at 70% VO2 max speed at the beginning and the end of the training, 3 times a week for 32 weeks), and resistance group (R, *n* = 40, 46 min per day, 3 days per week, with a 30% maximum load on a treadmill with a slope of 35°, 15 m/min). The soleus muscles were collected for analysis at baseline and every 8 weeks. Aging resulted in decreased soleus muscle mass and Bcl-2 levels in the mitochondria, while the levels of reactive oxygen species (ROS) and Bax did not change. HIIT reversed the age-associated activation of pro-apoptotic processes, but RT did not. In addition, when rats were aged from 8 to 16 months, the level of Cyt-C did not change, the Caspase-9 levels and Caspase-3 levels decreased gradually in the soleus muscles, the rats of both the HIIT and RT groups had these indices decreased at 32 weeks. The results suggest that the age-associated loss of muscle mass was reversed by training, and the effect of RT was better than that of HIIT. Both the HIIT and RT rats showed a decrease in the apoptosis of skeletal muscle cells after 32 weeks of intervention. HIIT performed better for long-term intervention regarding the pro-apoptotic factors. This study warranted further research to delineate the underlying mechanism of effects of different exercise methods on the changes of aging skeletal muscle at *in vivo* level.

## Introduction

Populations are rapidly aging worldwide. Data from the World Health Organization on aging and health projects show that the population over the age of 60 will double from 11%, in 2000, to 22% by 2050, increasing from 605 million to 2 billion (Organization, 2016). Due to the dramatic growth of the elderly population, the proportion of the elderly in society is increasing; therefore, the issue of aging is of great concern globally.

Sarcopenia caused by aging is an important factor leading to a decline in the quality of life of older people (Cosquéric et al., 2006; Swan et al., 2021). Sarcopenia is the muscle failure associated with aging. Studies have shown that exercise is an effective way to delay sarcopenia, which can be achieved by balancing skeletal muscle synthesis and catabolism, improving the skeletal muscle mitochondrial density and activity, reducing apoptosis, among other ways (Ziaaldini et al., 2017).

Apoptosis in muscle atrophy accelerates the process of muscle loss in older populations, which may be the key mechanism leading to muscle performance impairment (Dupont-Versteegden, 2005; Marzetti et al., 2013; Faitg et al., 2017). With aging, the mitochondrial-mediated pathways may induce apoptosis in skeletal muscle, playing an important role in sarcopenia (Dirks & Leeuwenburgh, 2002; Marzetti & Leeuwenburgh, 2006; Song et al., 2006; Kob et al., 2015; Ziaaldini et al., 2015). The over-opening of the mitochondrial permeability transition pore (MPTP) leads to the release of many pro-apoptotic proteins into the cytoplasm, such as cytochrome C (Cyt-C). It forms an apoptotic body with apoptosis protease-activating factor-1 and caspase-9. The apoptotic body causes caspase-9 to transform into Caspase-9 and to activate Caspase-3, causing apoptosis (Feldstein and Gores, 2005). Caspase-3 can degrade the actomyosin complex, and the degraded products are degraded by other protein systems in cells, resulting in a decline in skeletal muscle mass and strength (Du et al., 2004).

Mitochondrial caspase-dependent apoptosis (endogenous apoptosis) is regulated by Bcl-2 family proteins. Bcl-2 and Bcl-XL are anti-apoptotic proteins, and Bax is a pro-apoptotic protein (Ashkenazi et al., 2017). In addition, ROS are closely related to “oxidative stress.” A large amount of oxidative stress produces high levels of ROS, which breaks the balance between oxidation and antioxidants, damages the genetic material, changes the permeability of the mitochondrial membrane, and then induces apoptosis (Schieber and Chandel, 2013). Compared with young individuals, the expression of endogenous apoptosis-related proteins is increased in the skeletal muscle of older individuals (Dirks and Leeuwenburgh, 2002; Marzetti and Leeuwenburgh, 2006; Song et al., 2006; Kob et al., 2015; Ziaaldini et al., 2015).

So far, most of the research has mainly focused on the changes in endogenous apoptosis after sarcopenia transformation, and there is a lack of research on the temporal changes of the proteins related with the endogenous apoptosis pathway during aging. Compared with traditional exercise intervention methods, such as aerobic exercise and RT, HIIT, which is a new training method, is characterized by alternating short cycles of intense exercise with less intense periods of recovery (Bartlett et al., 2011; Heinrich et al., 2014). On role of physical activity in sarcopenia, many studies have shown that resistance exercise, aerobic exercise and HIIT can delay sarcopenia (Luo et al., 2013; Li et al., 2019; Neto et al., 2020). At present, there have been many studies on the effects of HIIT and aerobic exercise on skeletal muscle (Chavanelle et al. Sci Rep 2017; Martinez-Huenchullan et al., 2018; Martinez-Huenchullan et al., 2019). However, there are still are lacunae in the literature on role of compare different physical activity in sarcopenia, especially, research on the difference in the effects of RT and HIIT in sarcopenia on the aging process is rare. Therefore, In present study, the natural aging model of 32 weeks rats was established, and the rats were intervened with HIIT and RT during the aging process. The materials were taken every 8 weeks to observe the morphological changes of skeletal muscle and the changes of cytochrome c, caspase-9 and caspase-3 activities in the caspase dependent apoptosis pathway mediated by mitochondria of skeletal muscle cells, And the changes of Bcl-2 protein, Bax protein and ROS affecting caspase apoptosis pathway. The aim of the present study was to explore the effects of different exercise methods on the changes in the endogenous apoptotic pathway in the process of aging in order to provide a theoretical basis for exercise to delay the degeneration of aging skeletal muscle by regulating the endogenous apoptotic pathway. It was hypothesized that both HIIT and RT could both effectively reduce sarcopenia during aging, and improve endogenous apoptosis signaling pathway. And for endogenous apoptosis the effect of HIIT may be batter.

## Materials and Methods

### Experimental Animals

All experimental protocols were approved by the Institutional animal care and use committee of the Beijing Sport University (Ref. No: 2019026A). A total of eight-month-old male Sprague-Dawley rats (*N* = 130), weighing 650–700 g, were provided by Sipeifu Biotechnology (Beijing, China). According to Sengupta’s research, eight-month-old rats are approximately equivalent to twenty-year-old humans (Sengupta, 2013). After 1 weeks of acclimatization to the laboratory environment, 10 rats were randomly selected and sacrificed as the baseline group. Remaining rats were randomly divided into control group (C), HIIT group (H) and resistance group (R). Each group contained 40 rats.

Rats were given free access to standard food (includes water ≤10%, protein ≥18%, fat ≥4%, fiber ≤5%, fiber ≤8%, calcium ∼1.4%, phosphorus ∼0.8%) and water in the animal room of the Beijing Sport University (Certificate no. JDXT0029). The temperature of the animal room was 25 °C, with alternating light/dark cycles every 12 h (Specific Pathogen Free, SPF). Group C was fed for 32 weeks without exercise intervention; group H received HIIT intervention, and group R received RT intervention, which also lasted for 32 weeks.

### Training Protocol

Rats of group H performed a maximal oxygen uptake test before the intervention and every 4 weeks subsequently, in order to determine and appropriately adjust the speed of the treadmill for the HIIT. The tests were performed using an OxyMax Deluxe system (Columbus Instruments, USA). The rats were subjected to treadmill running at a speed corresponding to 70% VO2max for 5 min. Then, six loops of 3 min at 90% and 3 min at 50% VO2max speed treadmill running. Then, finishing with a speed corresponding to 70% VO2max for 5 min. HIIT was conducted for 32 weeks, 3 days per week, with each training session lasting for 46 min.

RT was performed on a treadmill (Weng et al., 2013) at a speed of 15 m/min and a slope of 35°. Rats were outfitted with a specially designed vest with 30% of max-weight bearing. The weight was adjusted according to the maximum weight-bearing capacity of rats, which was tested before intervention and every 4 weeks subsequently, to avoid adaptation to the intervention. Rats’ max-weight bearing capacity were the weight they could barely moving on a treadmill, at a speed of 15 m/min and a slope of 35°.The training plan for group R is shown in Table 1. RT lasted for 32 weeks, 3 days per week, with each training session lasting for 46 min.

**TABLE 1:** Training plan for RT.

Program	Training content
1	Weight bearing run for 15 s
2	Rest for 30 s
3	Repeat program 1–2 four times and rest for 3 min
4	Repeat program 3 three times and rest for 10 min
5	Repeat program 3 three times and finish the training

### Tissue Collection and Preservation

Before the training intervention, 10 rats were randomly selected, fasted for 24 h, and euthanized as the baseline group. After beginning the exercise intervention, every 8 weeks, 10 rats (if there was no mortality) in each group were rested and fasted for 24 h and were then euthanized to provide experimental samples. The rats were weighed and anesthetized using an intraperitoneal injection of 2% pentobarbital sodium (50 mg/kg). The soleus muscles of both legs were stripped and weighed, and the proximal fragment was used for the cross-sectional area (CSA) and the distal part was used for the ROS level and western blot tests.

### Soleus Muscle Mass Index Analyses

At the time of sampling, the bilateral soleus muscles of rats were stripped and weighed, and the SMI was obtained by dividing the sum of the bilateral soleus muscles of each rat by the corresponding rat body weight.

### CSA Analyses

The muscles were immersed in a paraformaldehyde stationary solution (Cat. No. G1101, Servicebio, China) for 24 h. Paraffin sections were prepared from the tissues soaked in the fixative, and then a H&E Staining Kit (Cat. No. G1005, Servicebio, China) was used for H&E staining. After taking pictures of the slices using a microscope (Nikon, Japan), a caseviewer (3DHISTECH, Germany) was used to scan the pictures. Five fields of view were selected from the center and four corners of each slice and saved in the TIF format. Image pro Plus6.0 (Media Cybernetics, Inc. United States) was used to calculate the skeletal muscle area in each visual field, which was then divided by the number of skeletal muscle fibers in the visual field to obtain the CSA.

### Muscle ROS Detection

The total protein in the muscle tissue was quantified using a protein assay kit (Thermo Fisher Scientific, United States). ROS levels were measured using kits from Jianglai Biotechnology (Cat. No. JL21051) using an enzyme-linked immunosorbent assay. After the muscle tissues were stored at 4°C, PBS was added and the mixture was homogenized. After centrifugation at 4°C and 5,000 × *g* for 10 min, the supernatants were aspirated. Subsequently, the tests were performed according to the manufacturer’s instructions.

### Extraction of Mitochondria From Skeletal Muscle

A tissue mitochondria isolation kit (Beyotime Biotechnology, China, Cat. No. C3606) was used to extract the mitochondria from the skeletal muscle. We weighed 50 mg of soleus muscle and washed once with 600 μl of PBS. The soleus muscle was placed in a centrifuge tube, minced with ophthalmic scissors on ice, 1 ml of PBS was added to the centrifuge tube and ice bathed for 3 min. Put the centrifuge tube into a low temperature centrifuge, centrifuge at 600 × *g* at 4°C for 10–20 s, and discard the supernatant. Add 800 μl of trypsin digestion solution to the centrifuge tube, ice bath for 20 min, put the centrifuge tube into a low temperature centrifuge at 4°C and centrifuge at 600 × *g* for 10–20 s, and discard the supernatant. Add 200 μl of separation reagent to the centrifuge tube, resuspend the tissue, put the centrifuge tube into a low temperature centrifuge at 600 × *g* for 10–20 s at 4°C, and discard the supernatant. 800 μl of separation reagent and 8 μl of PMSF were added to the centrifuge tube, and homogenized with a homogenizer. Put the centrifuge tube into a low temperature centrifuge and centrifuge at 600 × *g* for 5 min at 4°C, take the supernatant and transfer it to another centrifuge tube. Put the centrifuge tube into a low temperature centrifuge and centrifuge at 3,500 × *g* at 4°C for 10 s, discard the supernatant, and the precipitate is mitochondria.

### Western Blot Analysis

The total protein in the muscle tissue and muscle mitochondria tissue was extracted and quantified using a protein assay kit (Thermo Fisher Scientific, United States). Proteins were separated on 15 wells of 12% SDS-PAGE gels, 20 μg in each well, by electrophoresis. The proteins were then transferred onto polyvinylidene fluoride (PVDF) membranes. Using Bovine serum albumin (BSA) as the blocking reagent and the target proteins were blocked and probed overnight at 4 °C using a Bax antibody (1:1,000, Cat. No. 2772T, CST, United States), Bcl-2 (1:4,000, Cat. No. ab196495, Abcam, United States), Cyt-C (1:5,000, Cat. No. ab133504, Abcam, United States), Caspase-3 (1:1,000, Cat. No. 9662S, CST, United States), Caspase-9 (1:2,000, Cat. No. ab184786, Abcam, United States), GAPDH (1:3,000, Cat. No. ab9485, Abcam, United States), and COXIV (1:2,000, Cat. No. 4850, CST, United States). All primary antibodies are from Rabbit. The following day, after washing with a TBST solution three times for 10 min each, the membranes were incubated with goat horseradish peroxidase (HRP)-conjugated goat anti-rabbit IgG (1:10,000, Cat. No. ab205718, Abcam, United States) at 25°C for 1 h. The membranes were washed six times with TBST for 5 min each. Signals were detected using an enhanced chemiluminescence (ECL) reagent. All bands were analyzed semi-quantitatively using ImageJ software and Total Lab Quant V11.5 (Newcastle upon Tyne, United Kingdom).

### Statistical Analyses

Statistical analysis was performed using SPSS 22.0 (IBM SPSS Statistics, Armonk, NY, United States). All data are presented as mean ± SEM. All indices were analyzed using two-way ANOVA, time and exercise patterns were assessed as independent variables. The significance level was set at *p* < 0.05.

## Results

### Skeletal Muscle Morphology and Weight

**FIGURE 1:**
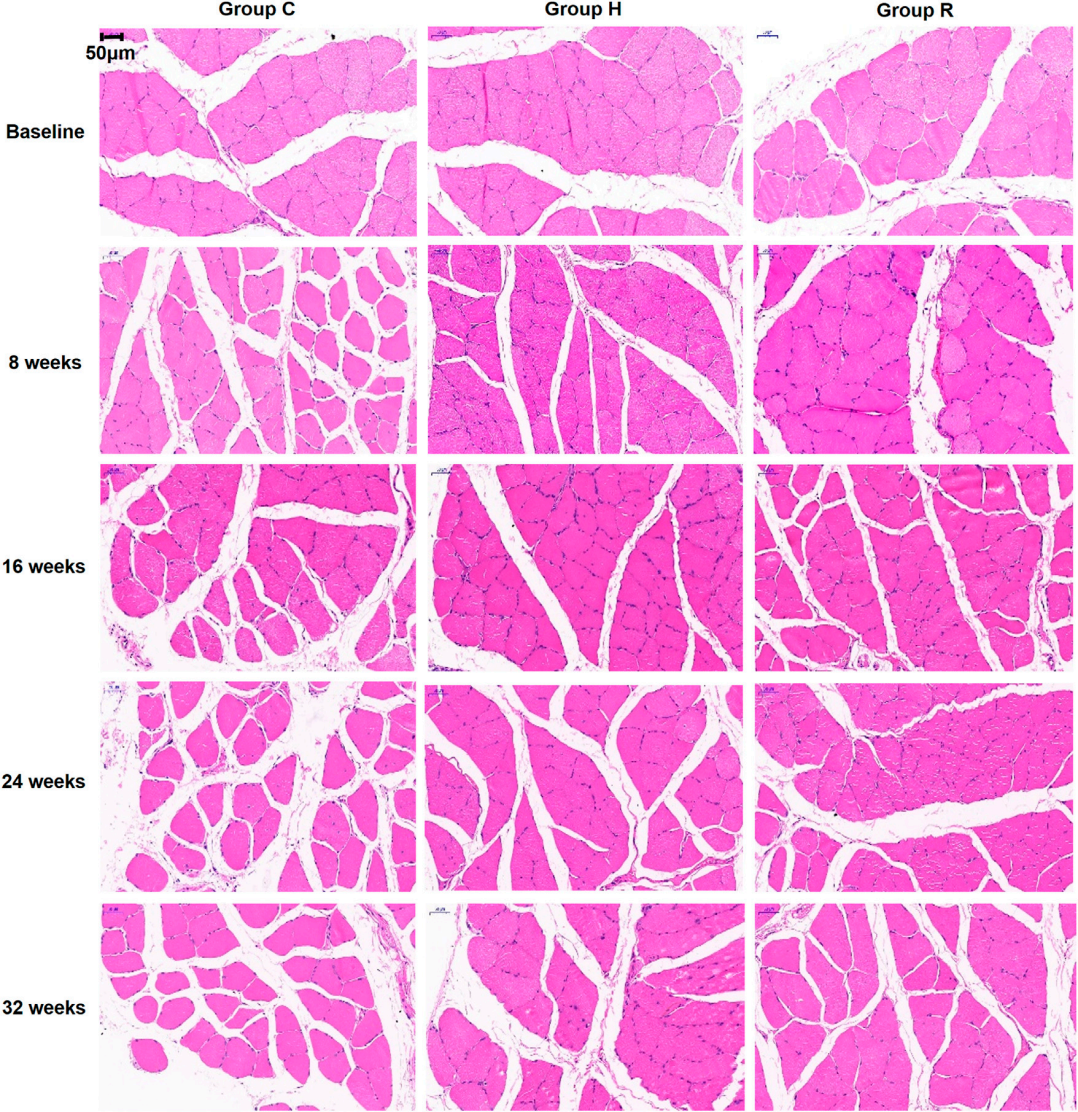
HE staining sections of soleus muscle of rats in each group were observed under 400 times microscope.

**FIGURE 2:**
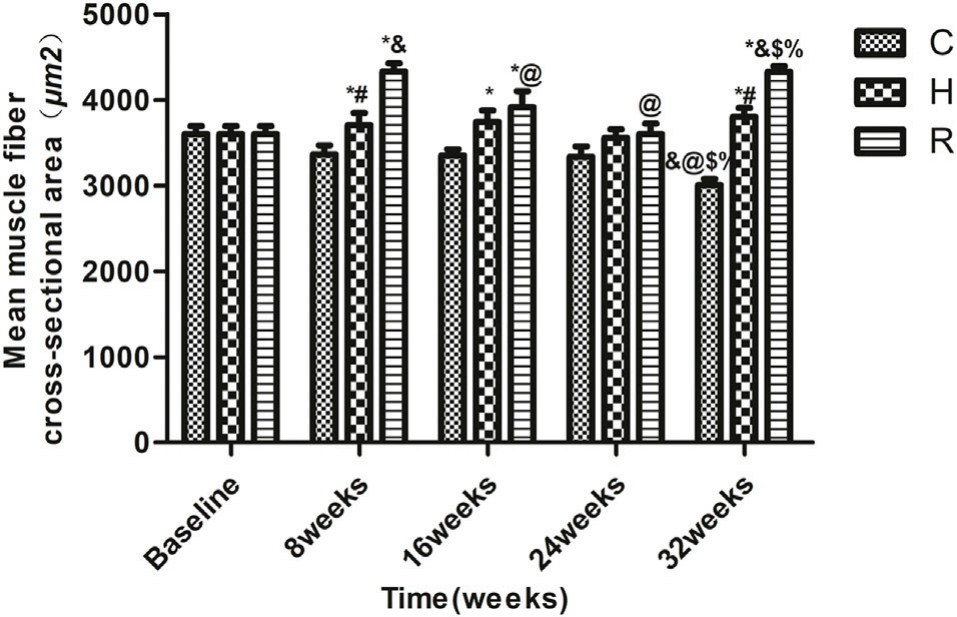
The soleus muscle fiber CSA of rats in each group. * Significant different from C and other group; ^#^ Significant different from H and R; & Significant different from Baseline and other weeks; @ Significant different from 8 weeks and other weeks; $ Significant different from 16 weeks and other weeks; % Significant different from 24 weeks and other weeks (*p* < 0.05).

The results, as shown in Figure 1, indicate that age-related muscle fiber CSA loss occurred at 32 weeks in group C (*p* < 0.001). The muscle fiber CSAs of groups H and R were higher than those of group C at 8, 16, and 32 weeks (*p* < 0.05) and that of group R was higher than that of group H at 8 and 32 weeks (*p* < 0.001). At 8 and 32 weeks, the muscle fiber cross-sectional area of the R group rats was higher than at other time points (*p* < 0.05) (Figure 1, ×400 magnification; Figure 2).

**FIGURE 3:**
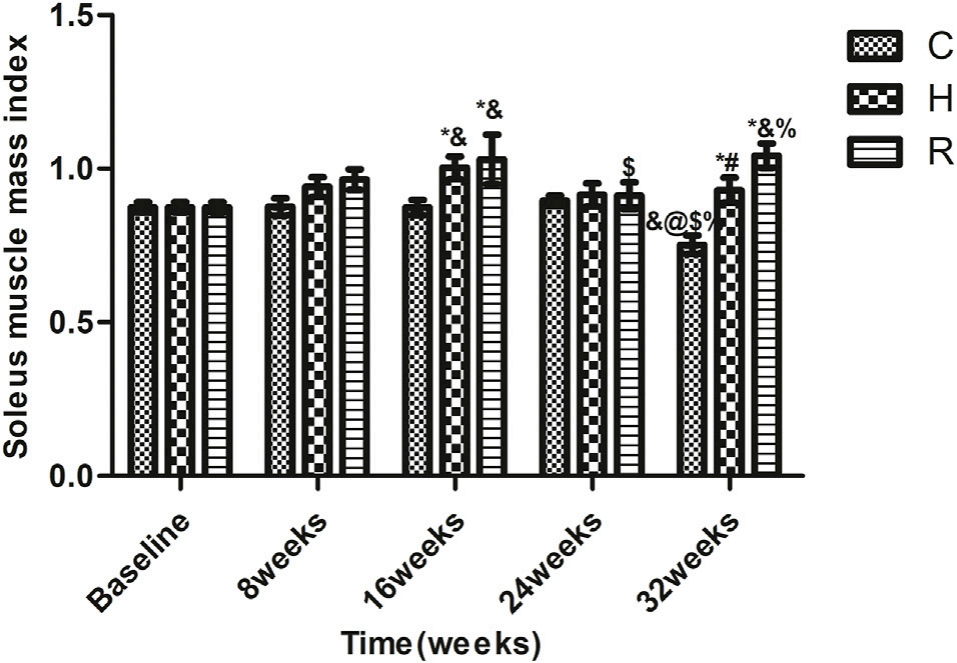
The soleus muscle mass index of rats in each group. * Significant different from C and other group; ^#^ Significant different from H and R; & Significant different from Baseline and other weeks; @ Significant different from 8 weeks and other weeks; $ Significant different from 16 weeks and other weeks; % Significant different from 24 weeks and other weeks (*p* < 0.05).

Age-related SMI loss occurred at 32 weeks in group C (*p* < 0.05). The SMI values of the rats of groups H and R were higher than those of group C rats at 16 and 32 weeks (*p* < 0.05) and those of group R rats were higher than those of group H rats at 16 and 32 weeks (*p* < 0.05). The SMI values of group H rats were higher than the baseline level at 16 weeks (*p* < 0.05), and those of group R were higher at 32 weeks than the baseline level and the levels at 24 weeks (*p* < 0.05) (Figure 3).

### Factors Affecting Apoptosis

**FIGURE 4:**
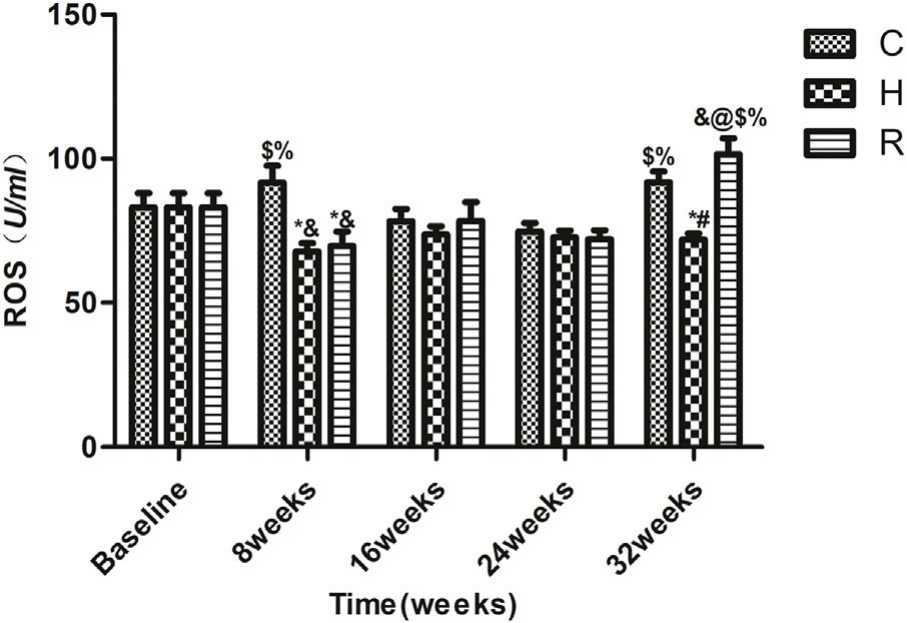
The ROS level of soleus muscle in each group. * Significant different from C and other group; # Significant different from H and R; & Significant different from Baseline and other weeks; @ Significant different from 8 weeks and other weeks; $ Significant different from 16 weeks and other weeks; % Significant different from 24 weeks and other weeks. (*p* < 0.05).

The level of ROS in group C rats at 8 and 32 weeks was higher than that at 16 and 24 weeks (*p* < 0.05). The ROS levels of H group decreased significantly at 8 weeks of training (*p* < 0.05) and remained stable during the 8–32 weeks period. Those of group R decreased significantly at 8 weeks (*p* < 0.05) but increased significantly at 32 weeks (*p* < 0.001). The ROS levels of groups H and R were significantly lower than those of group C at 8 weeks (*p* < 0.001), but only those of group H were lower than those of the other groups at 32 weeks (*p* < 0.001) (Figure 4).

**FIGURE 5:**
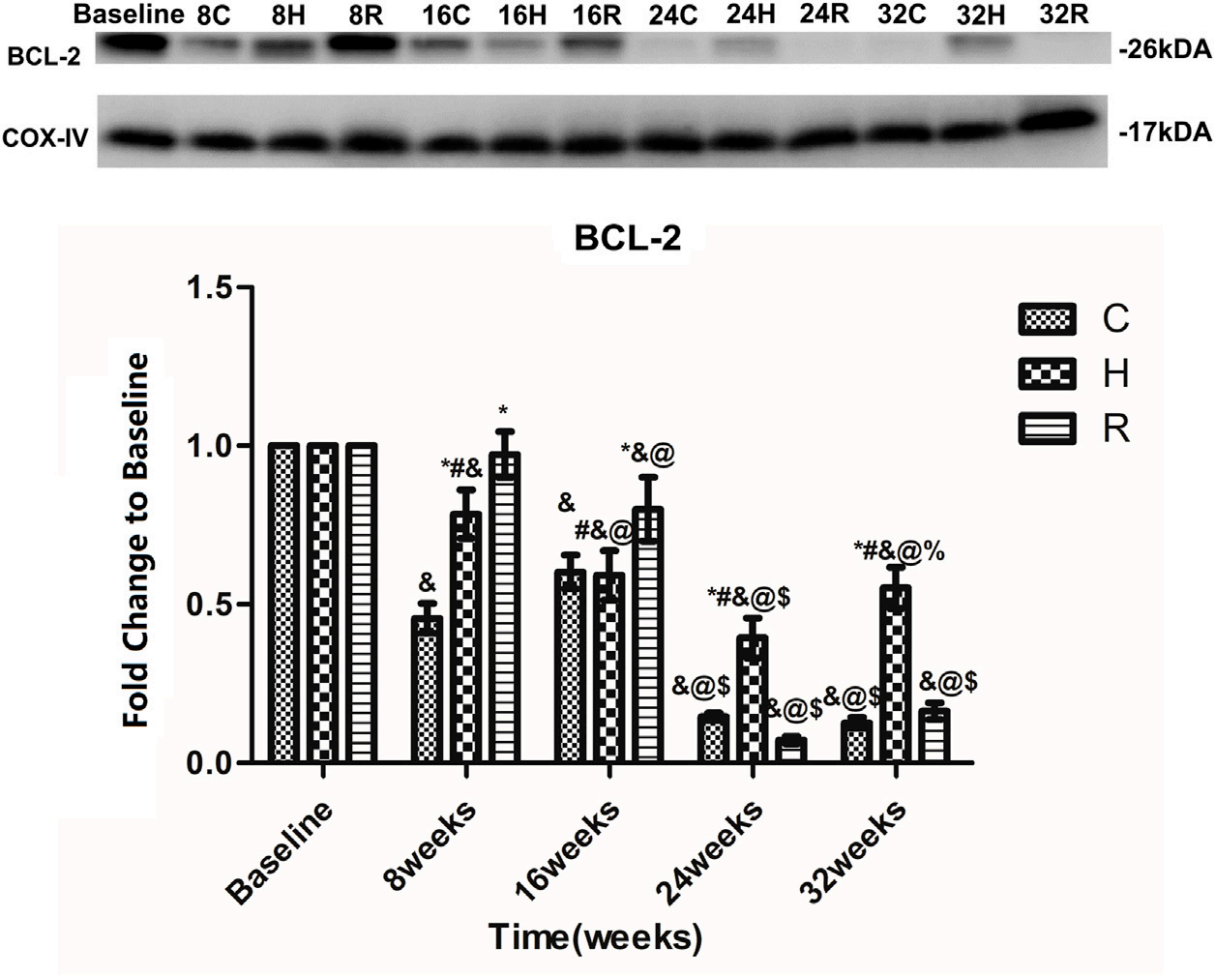
Age and exercise training effects on Bcl-2 levels in mitochondria of soleus muscle, were evaluated by Western blot. * Significant different from C and other group; # Significant different from H and R; & Significant different from Baseline and other weeks; @ Significant different from 8 weeks and other weeks; $ Significant different from 16 weeks and other weeks; % Significant different from 24 weeks and other weeks. (*p* < 0.05).

The levels of Bcl-2 in the mitochondria of the soleus muscle in group C decreased significantly at 8 and 24 weeks (*p* < 0.001), and showed a gradual downward trend. The Bcl-2 levels in group H showed a downward trend in the 8–24 weeks period of training, but increased at 32 weeks (*p* < 0.05). It is apparent from this table that the Bcl-2 levels in group H were significantly higher in groups C and R at 24 and 32 weeks of training (*p* < 0.001). In addition they were decreased in group R at 16 and 24 weeks of training (*p* < 0.05), and were higher than in other groups at 8 and 16 weeks (*p* < 0.05) but were equal to the baseline value at 24 and 32 weeks (*p* < 0.05) (Figure 5).

**FIGURE 6:**
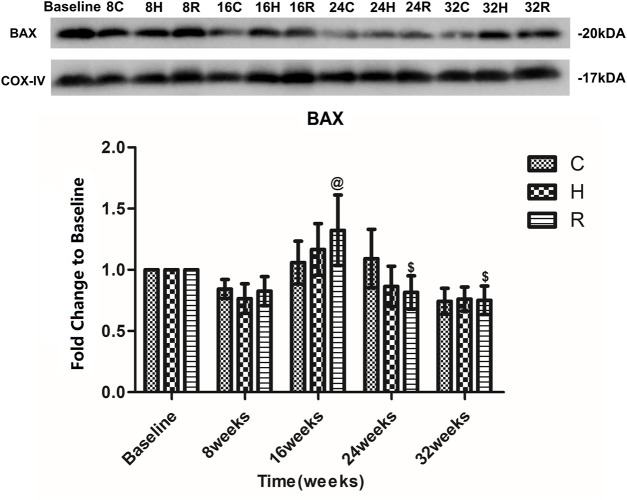
Age and exercise training effects on BAX levels in mitochondria of soleus muscle, were evaluated by Western blot. @ Significant different from 8 weeks and other weeks; $ Significant different from 16 weeks and other weeks. (*p* < 0.05).

The level of Bax in the mitochondria of the soleus muscle in group C increased at 16 and 24 weeks, and decreased at 32 weeks, but there was no significant difference at each time point (*p* > 0.05). The Bax level in group R increased at 16 weeks and decreased at 24 weeks (*p* < 0.05). Group H presented a similar Bax level trend as group R, but none of these differences were statistically significant (*p* > 0.05) (Figure 6).

### Endogenous Apoptotic Protein

**FIGURE 7:**
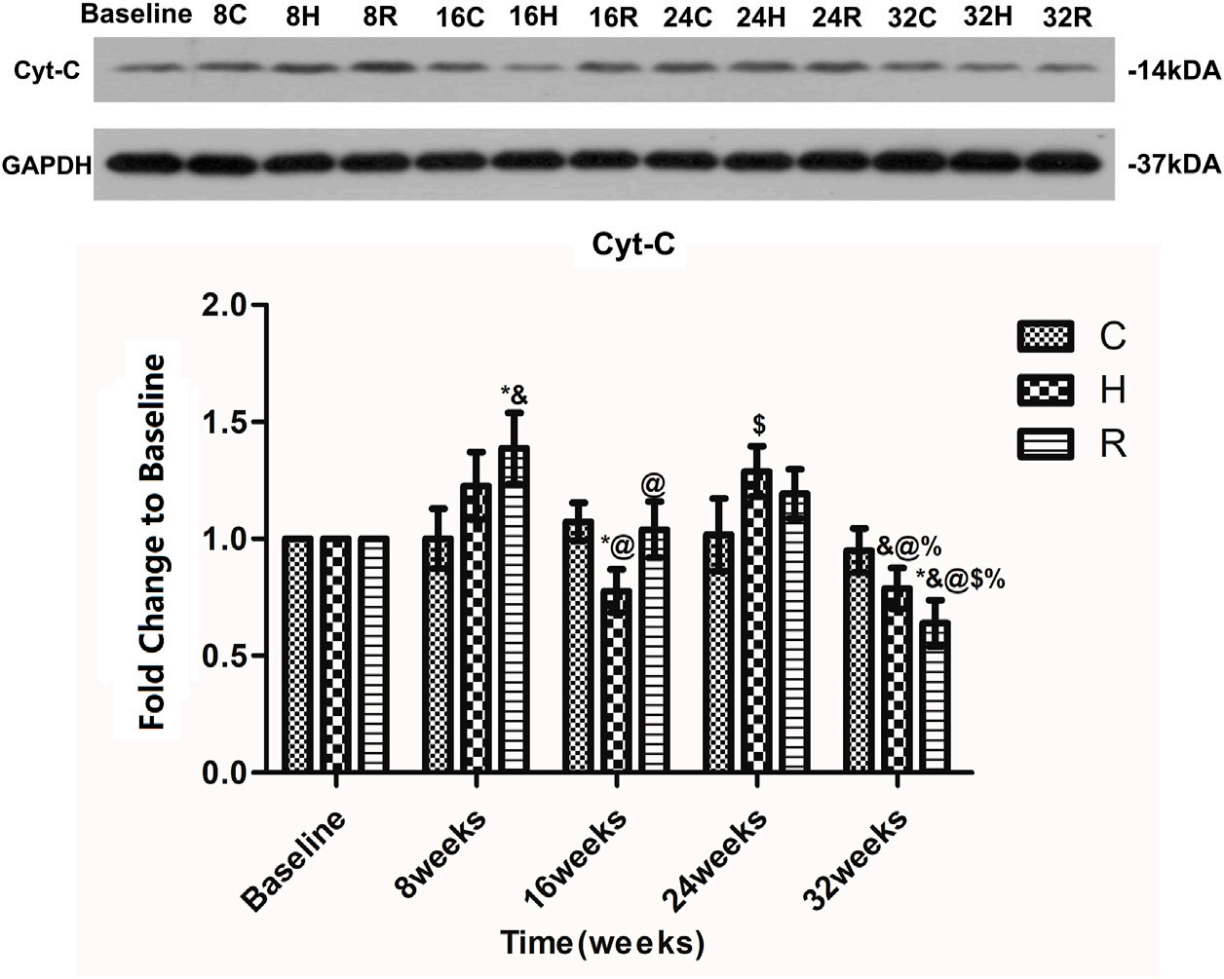
Age and exercise training effects on Cyt-3 levels in mitochondria of soleus muscle, were evaluated by Western blot. * Significant different from C and other group; & Significant different from Baseline and other weeks; @ Significant different from 8 weeks and other weeks; $ Significant different from 16 weeks and other weeks; % Significant different from 24 weeks and other weeks. (*p* < 0.05).

There was no age-related change in the level of Cyt-C protein in the soleus muscle of group C at 32 weeks of aging. The Cyt-c level increased significantly in group H at 24 weeks (*p* = 0.001) and decreased significantly at 16 and 32 weeks (*p* < 0.05) and that of group R increased significantly at 8 weeks and decreased significantly at 16 and 32 weeks (*p* < 0.05). The Cyt-c level in group H was significantly lower than that in group C at 16 weeks (*p* < 0.05), and that of group R was significantly higher than that of group C at 8 weeks, but was significantly lower than that of group C at 32 weeks (*p* < 0.05) (Figure 7).

**FIGURE 8:**
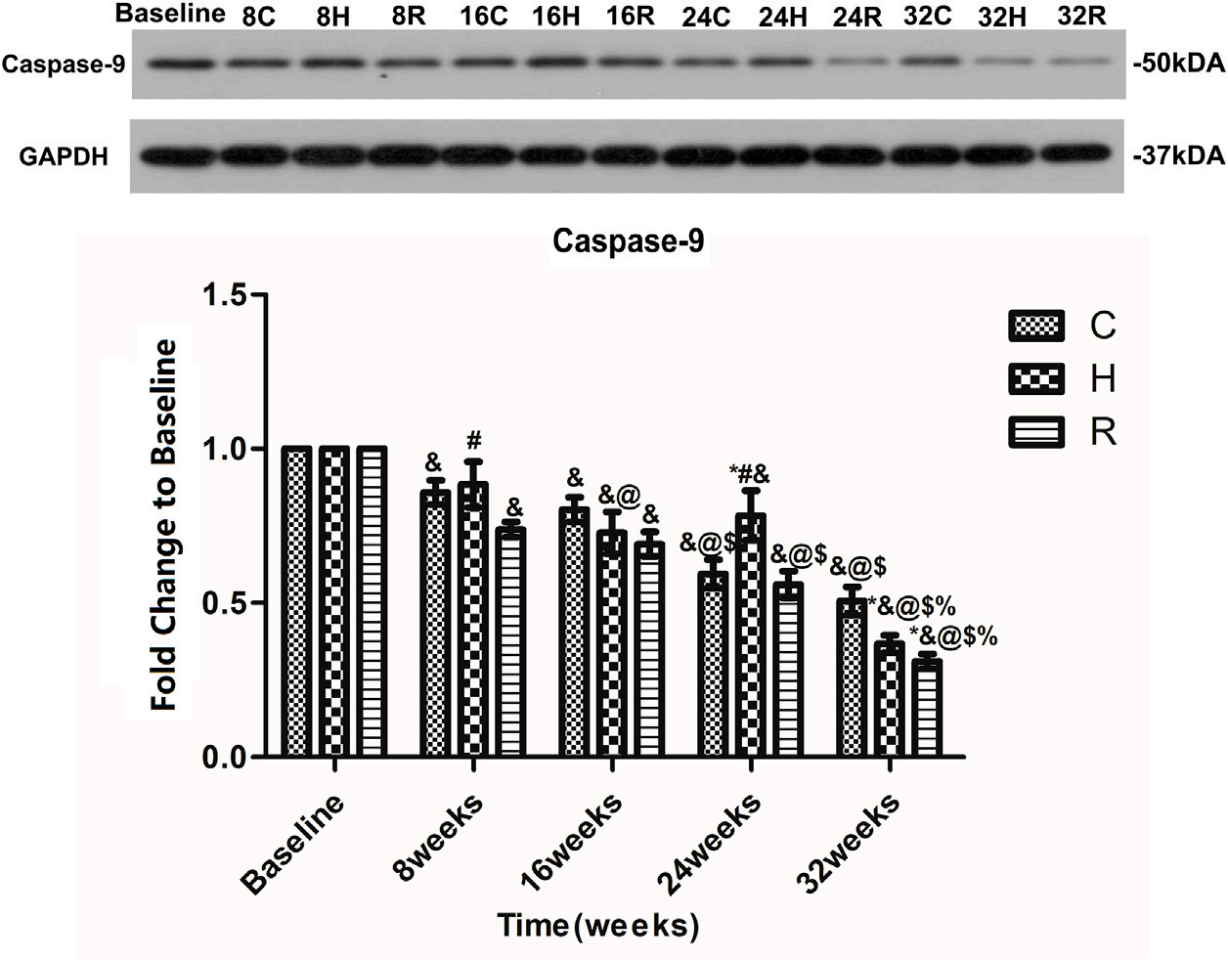
Age and exercise training effects on Caspase-9 levels in mitochondria of soleus muscle, were evaluated by Western blot. * Significant different from C and other group; # Significant different from H and R; & Significant different from Baseline and other weeks; @ Significant different from 8 weeks and other weeks; $ Significant different from 16 weeks and other weeks; % Significant different from 24 weeks and other weeks. (*p* < 0.05).

An age-related decrease in the caspase-9 level in the soleus muscle was observed in all groups. Interestingly, the decrease in H group was observed to occur mainly at 16 and 32 weeks (*p* < 0.05). At 16 weeks, the caspase-9 protein level in group H was higher than that of group R (*p* < 0.05). At 24 weeks, that of group H was higher than those of groups C and R (*p* < 0.05). However, at 32 weeks, those of groups H and R were lower than those of group C (*p* < 0.05) (Figure 8).

**FIGURE 9:**
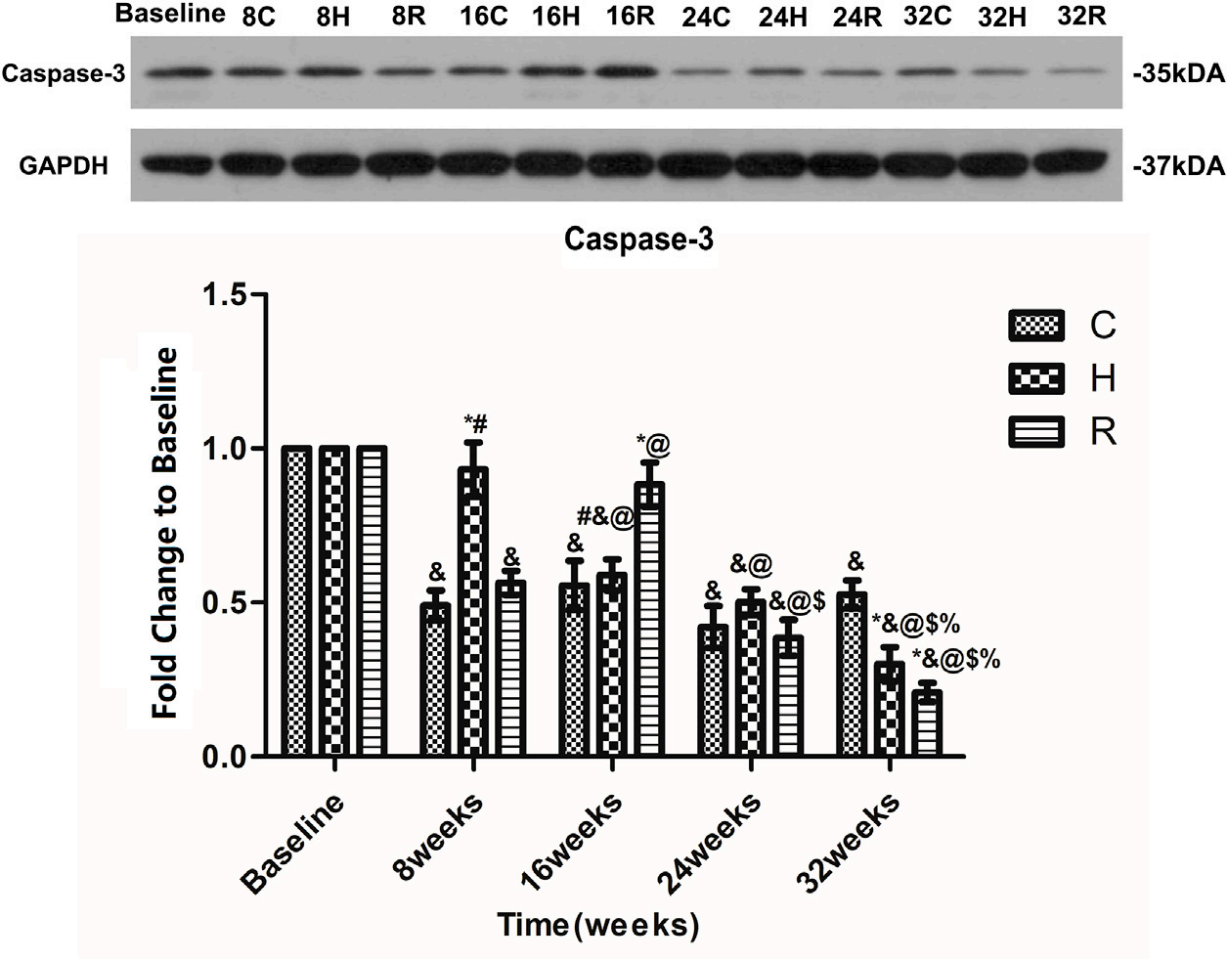
Age and exercise training effects on Caspase-3 levels in mitochondria of soleus muscle, were evaluated by Western blot. * Significant different from C and other group; # Significant different from H and R; & Significant different from Baseline and other weeks; @ Significant different from 8 weeks and other weeks; $ Significant different from 16 weeks and other weeks; % Significant different from 24 weeks and other weeks. (*p* < 0.05).

The level of caspase-3 in the soleus muscle in group C decreased at 8 weeks (*p* < 0.001), and then remained stable. In group H, the level of Caspase-3 in the soleus muscle decreased with age. It was significantly higher than that of groups C and R at 8 weeks, but lower than that of group C at 32 weeks (*p* < 0.001). In group R, the caspase-3 level increased at 16 weeks and decreased at other time points (*p* < 0.001), being significantly higher than that of groups C and H at 16 weeks, but lower than that of group C at 32 weeks (*p* < 0.001) (Figure 9).

## Discussion

In this study, through continuous observation of naturally aging rats, it was confirmed that the decrease in the muscle mass index and in the CSA of the soleus muscle in naturally aging rats was the earliest at 16-months-old of aging, which updated the time of age-related muscle atrophy. Several reports have shown that the SMI of rats over 24 months of age is significantly lower than that of rats aged 4–6 months (Rice et al., 2005; Song et al., 2006; Liu, 2018). The earliest age-related decrease in the soleus CSA has occurred at 25 months of age (Wang et al., 2015). Most of these studies are cross-sectional studies, which cannot demonstrate when age-related atrophy of the rat soleus muscle occurs. In this study, a longitudinal observation of aging rats (8–16-months-old) was conducted to explore the problem and yielded different results, which filled the gap in existing research.

Exercise can improve the age-related atrophy of muscles (Cui et al., 2021; Luo et al., 2013; Zhao et al., 2016; Ribeiro et al., 2017; Li et al., 2019; Neto et al., 2020); however, most current studies have been performed on elderly rats undergoing 12 weeks of exercise training, in comparison with the skeletal muscle of the control group rats. The results of this study suggest that both HIIT and RT can effectively increase the SMI of soleus muscle in rats after 16 and 32 weeks of training, and both methods can effectively increase the CSA of the rat soleus muscle at 8 and 32 weeks of training. One interesting finding is that the increase of SMI and CSA caused by exercise occurred at different times. However, this result has not previously been described. It may support the hypothesis that exercise induced increases in muscle fiber number (Gonyea et al., 1986). In addition, the muscle enhancement effect of 32 weeks of RT intervention was better than that of HIIT, which may be because RT can better activate the pathways related with muscle synthesis (Ribeiro et al., 2017; Neto et al., 2020).

In conclusion, the earliest age-related loss of the soleus muscle occurred at 16 months of age, a 32-weeks exercise intervention can improve the aging atrophy of the soleus muscle in rats, and the effect of RT is better than that of HIIT.

High concentrations of ROS can damage the outer membrane of mitochondria, leading to MPTP, which increases the release of Cyt-C to promote apoptosis (Schieber and Chandel, 2013). Many studies have discussed the age-related increase in ROS levels (Muller et al., 2007; Sullivan-Gunn and Lewandowski, 2013; Ziaaldini et al., 2015; Boengler et al., 2017; Damiano et al., 2019; Jiang, 2019), but it is uncertain when this phenomenon first occurs. In this study, in contrast to other studies, however, there were no differences in the ROS levels between each time point and the corresponding baseline value in the control group. This may be due to the different strains and living environments of the rats. In addition, this study found that 32 weeks of HIIT reduced the ROS levels. These relationships may partly be explained by HIIT improves the ability of the skeletal muscle to scavenge ROS and the mitochondrial respiration ability or other pathway (Alhadlaq et al., 2019; Chrois et al., 2019). Although many studies have shown that exercise can reduce the ROS levels (Vezzoli et al., 2019; Bartlett et al., 2020), in this study, 32 weeks of RT intervention increased the ROS levels. It is speculated that a higher exercise intensity of RT leads to oxidative stress (Liu et al., 2008) and ROS accumulation at 32 weeks. This indicates that HIIT may be a better intervention option for reducing the ROS levels.

Bax, which is a pro-apoptotic protein, can also induce MMPT. This can lead to the release of Cyt-C and, ultimately, promote apoptosis. Bcl-2 can reduce apoptosis by inhibiting the activity of Bax (Ashkenazi et al., 2017; Hikita and Takehara, 2017). This study confirmed that the Bcl-2 level in the mitochondria of skeletal muscle cells decreases with aging, and that the Bax level does not change with aging. Many studies have focused on the aging changes in the Bcl-2 and Bax levels, but the results are contradictory (Dirks and Leeuwenburgh, 2002; Baker and Hepple, 2006; Song et al., 2006; Liao et al., 2017). In general, the gene expression of Bcl-2 and Bax in the skeletal muscle decreases with aging. The protein expression of Bcl-2 in skeletal muscle showed an age-related decrease, whereas the expression of Bax showed an opposite trend. In skeletal muscle mitochondria, the expression of these proteins does not change with age. In this study, we not only found an age-related decrease in the Bcl-2 level in skeletal muscle mitochondria, but also found that it first occurred at 10 months of age. What is surprising is that this time point is much earlier than that observed in other studies, suggesting that the age-related changes in the levels of Bcl-2 family proteins in skeletal muscle mitochondria may occur earlier. Unfortunately, there were no age-related changes in Bax expression. In addition, some studies found that exercise training resulted in adaptations in the apoptotic signaling by Bcl-2 family proteins in the skeletal muscle of old rats (Song et al., 2006; Lin et al., 2013; Liao et al., 2017; Li et al., 2019); however, they did not compare the effect of HIIT and RT on the Bcl-2 levels. We found that RT was more effective before 16 weeks of aging, and that HIIT was more effective after the 16-weeks time point. This finding, while preliminary, suggests that HIIT is a more suitable long-term form of exercise for increasing the Bcl-2 level in skeletal muscle mitochondria. It is worth noting that RT can significantly increase the expression of Bcl-2 in the first 16 weeks (12-months-old), which may be the reason why the drastic changes in the early stage activated the feedback regulation of the body or HIIT activates other pathways (Ahamed et al., 2020)and, finally, caused it to lose its function after 16 weeks. In addition, the expression of Bax in mitochondria was not affected by exercise. The specific reasons and mechanisms require further studies. Overall, HIIT performed better as a long-term intervention regarding the ROS and Bcl-2 levels.

Cyt-C initially exists on the cristae of mitochondria and plays an important role in mitochondrial respiration. When the mitochondrial membrane permeability changes, Cyt-C is released into the cytoplasm and induces apoptosis. The release of Cyt-C is considered to be a marker of the activation of the mitochondrial apoptotic pathway (Dirks and Leeuwenburgh, 2002). We detected the level of Cyt-C in the rat soleus muscle every 8 weeks, and found that it remained unchanged during the 32 weeks of aging (8–16-months-old); some studies have drawn a similar conclusion (Dirks and Leeuwenburgh, 2002; Chung and Ng, 2006), but they only tested young and old rats. It is not yet clear when the aging-associated increase in the Cyt-C level occurs. It is worth noting that some studies found that, compared with 3-month-old rats, the content of Cyt-C in the skeletal muscle of 8-month-old rats increased (Ziaaldini et al., 2015) and, compared with 4-month-old rats, the content of Cyt-C in the skeletal muscle of 22-month-old rats increased (Kang et al., 2013). This study showed that there was no age-related change in the Cyt-C content in skeletal muscle from 8 to 16 months of age. According to these data, we can infer that the aging changes in the Cyt-C content in skeletal muscle may occur in rats under 8 months of age. We observed that 16- and 32-weeks HIIT interventions can reduce the release of Cyt-c. However, eight- and 24-weeks HIIT interventions increased the release of Cyt-C, which is consistent with the results of previous studies (Wang, 2018). This may be due to the fact that long-term HIIT intervention results in skeletal muscle adaptation regarding Bcl-2 and ROS in old rats. In addition, HIIT intervention can downregulate hist1h1c (Li et al., 2019) and improve the respiratory capacity of skeletal muscle mitochondria (Chrois et al., 2019), which can reduce the release of Cyt-c. RT can reduce the release of Cyt-C in the skeletal muscle of aged rats (Luo et al., 2013; Lin et al., 2014). We found that 32 weeks of RT intervention could achieve this effect. More importantly, at 32 weeks, the effect of HIIT was not as good as that of RT, in contrast with the results obtained for ROS and bcl-2. We speculate that RT can achieve this effect by changing the mitochondrial membrane potential and the levels of mitochondrial fusion protein (Su et al., 2020).

Caspase-9 can activate caspase-3 and promote apoptosis, and some studies have found an age-related increase in the caspase-9 level in skeletal muscle (Alway et al., 2002; Baker & Hepple, 2006). However, it is not known when it happens. In this study, no age-related increase in the caspase-9 level was found in the skeletal muscle of rats aged 8–16 months. Therefore, the specific time of this phenomenon requires further study. After 32 weeks of HIIT and resistance exercise intervention, the expression of caspase-9 decreased in the skeletal muscle of rats. The difference is that the caspase-9 level in the skeletal muscle of rats treated with HIIT from 8 to 24 weeks showed no significant changes, but it decreased at 32 weeks, which is similar to the trend observed for Cyt-C in group H. However, resistance exercise can cause the skeletal muscle mass of rats to decrease gradually during aging, which indicates that resistance exercise will play a bigger role in this in the beginning. But, the changes in the levels of Cyt-C and caspase-9 observed in group R were different during aging, indicating that resistance exercise may also reduce the level of caspase-9 through other factors (Mejías-Peña et al., 2017; Ribeiro et al., 2017; Neto et al., 2020), which requires further study.

This study confirmed that the content of Caspase-3 in the soleus muscle of aging rats decreased at 10-months-old and remained unchanged at 10–16- months-old. Some studies have shown that the level of Caspase-3 in skeletal muscle remains unchanged during aging (6-month-old rats were compared with 24-month-old rats) (Dirks and Leeuwenburgh, 2002; Chung and Ng, 2006). However, Some outcomes are contrary to that of Dirks et al. who found that compared with the 6–8-month-old rats, the level of caspase-3 was significantly increased in 27–35-month-old rats (Baker and Hepple, 2006; Song et al., 2006). Therefore, we speculate that the age-related increase in the level of Caspase-3 in the skeletal muscle of rats occurs after 24 months of age. In this study, the reason for the decrease in the caspase-3 level at the age of 10 months may be that the metabolism of cells in young rats is exuberant, and the cell renewal is fast; thus, apoptosis is active. At the age of 10–16 months, the metabolic rate decreases with the increase in age; therefore, the level of caspase-3 decreases and remains stable, compared with that at the age of 8 months (Ying and Liang, 2015). In addition, this study confirmed that 32 weeks of HIIT and RT intervention can reduce the level of Caspase-3 in the skeletal muscle of aging rats. However, in the aging process, HIIT can gradually reduce the caspase-3 level in skeletal muscle, while RT can increase and decrease the caspase-3 level. Therefore, the effect of HIIT appeared to be more stable than that of RT. We found that the results of the levels of caspase-9 and caspase-3 were consistent after 32 weeks of intervention. However, the changes in the Caspase-3 level in the three groups presented a different trend than that observed for the caspase-9 level during aging. It is speculated that aging and exercise may regulate the caspase-3 level through the death receptor pathway and endoplasmic reticulum pathway, and this process may affect the levels of Bad (BCL-XL/Bcl-2-associated death promoter), TNF-α, Caspase-8, caspase-12, and so on (Marzetti & Leeuwenburgh, 2006; Luo et al., 2013; Mejías-Peña et al., 2017; Ribeiro et al., 2017; Ahamed et al., 2018; Ahamed et al., 2021).

In conclusion, the level of Cyt-C in the soleus muscle remained unchanged, while the levels of caspase-9 and caspase-3 decreased during the 32 weeks of aging. Exercise training for 32 weeks reduced the level of Caspase-3 through Cyt-C and caspase-9. The effects of HIIT and RT were the same at 32 weeks.

## Conclusion

This study set out to find a better way to delay sarcopenia and explore the effects of different exercise methods on the changes in the endogenous apoptotic pathway in the process of aging. We demonstrated that the age-associated loss of muscle mass was reversed by training, and that the effect of RT was better than that of HIIT. There was no age-related increase in skeletal muscle apoptosis in 8–16-month old rats. However, both HIIT and RT reduced the apoptosis level of skeletal muscle cells after 32 weeks of intervention. HIIT performed better in long-term intervention regarding the pro-apoptotic factors, and there was no difference in the effect of HIIT and RT on apoptosis at 32 weeks. Although these results generally support the idea that exercise reduces skeletal muscle apoptosis in aged rats, we could not find the specific time of the age-associated increase in apoptosis; therefore, further studies aimed at observing the apoptosis of skeletal muscle for a longer period are required to assess this.

## Data Availability

The original contributions presented in the study are included in the article/Supplementary Material, further inquiries can be directed to the corresponding author.
